# Purification, kinetic characterization, and site-directed mutagenesis of *Methanothermobacter thermautotrophicus* RFAP Synthase Produced in *Escherichia coli*

**DOI:** 10.3934/microbiol.2019.3.186

**Published:** 2019-07-23

**Authors:** Matthew E. Bechard, Payam Farahani, Dina Greene, Anna Pham, Andrew Orry, Madeline E. Rasche

**Affiliations:** 1 Department of Pathology, Microbiology and Immunology, Vanderbilt University Medical Center, Nashville, TN 37232; 2 Chemistry and Biochemistry Department, California State University at Fullerton, 800 North State College Blvd., Fullerton, CA 92834; 3 Northern California Regional Laboratories, The Permanente Medical Group, Berkeley, CA 94710; 4 Molsoft L.L.C., 11199 Sorrento Valley Road, S209, San Diego, CA 92121

**Keywords:** methanogenesis, methanopterin, RFAP synthase, site-directed mutagenesis, substrate binding

## Abstract

Methane-producing archaea are among a select group of microorganisms that utilize tetrahydromethanopterin (H_4_MPT) as a one-carbon carrier instead of tetrahydrofolate. In H_4_MPT biosynthesis, β-ribofuranosylaminobenzene 5′-phosphate (RFAP) synthase catalyzes the production of RFAP, CO_2_, and pyrophosphate from *p*-aminobenzoic acid (*p*ABA) and phosphoribosyl-pyrophosphate (PRPP). In this work, to gain insight into amino acid residues required for substrate binding, RFAP synthase from *Methanothermobacter thermautotrophicus* was produced in *Escherichia coli*, and site-directed mutagenesis was used to alter arginine 26 (R26) and aspartic acid 19 (D19), located in a conserved sequence of amino acids resembling the *p*ABA binding site of dihydropteroate synthase. Replacement of R26 with lysine increased the *K_M_* for *p*ABA by an order of magnitude relative to wild-type enzyme without substantially altering the *K_M_* for PRPP. Although replacement of D19 with alanine produced inactive enzyme, asparagine substitution allowed retention of some activity, and the *K*_M_ for *p*ABA increased about threefold relative to wild-type enzyme. A molecular model developed by threading RFAP synthase onto the crystal structure of homoserine kinase places R26 in the proposed active site. In the static model, D19 is located close to the active site, yet appears too far away to influence ligand binding directly. This may be indicative of the protein conformational change predicted previously in the Bi-Ter kinetic mechanism and/or formation of the active site at the interface of two subunits. Due to the vital role of RFAP synthase in H_4_MPT biosynthesis, insights into the mode of substrate binding and mechanism could be beneficial for developing RFAP synthase inhibitors designed to reduce the production of methane as a greenhouse gas.

## 1. Introduction

**Figure 1.**
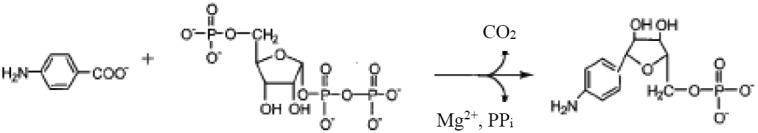
Reaction catalyzed by RFAP synthase.

β-Ribofuranosylaminobenzene 5′-phosphate (RFAP) synthase catalyzes the first committed step in the biosynthesis of tetrahydromethanopterin (H_4_MPT), a tetrahydrofolate-like cofactor originally discovered in methane-producing microorganisms (methanogens) [1]–[6]. The coenzyme H_4_MPT is essential not only for the microbial production of methane but also for the survival of all known methanogenic organisms [7]–[10]. RFAP synthase catalyzes the transfer of a phosphoribosyl group from phosphoribosylpyrophosphate (PRPP) to the aminobenzene moiety of *p*-aminobenzoic acid (*p*ABA), forming RFAP, CO_2_, and inorganic pyrophosphate (Figure 1). The decarboxylation of *p*ABA catalyzed by RFAP synthase gives H_4_MPT the main chemical feature that distinguishes it from tetrahydrofolate, which retains the carboxyl group attached to the benzene ring [11].

RFAP synthase is distinctive among phosphoribosyltransferases in that the phosphoribosyl moiety of the product is bound to a carbon atom rather than a nitrogen atom [1],[2]. Detailed kinetic analysis of RFAP synthase from the hyperthermophilic methanogen *Methanocaldococcus jannaschii* predicts an ordered Bi-Ter mechanism in which PRPP binds first, followed by *p*ABA [12]. During catalysis, the order of product release is CO_2_, RFAP, and then PP_i_. In the mechanism proposed by Dumitru and Ragsdale [12] the formation of this unusual bond involves the production of a negatively charged species at the C-1 position of *p*ABA. This carbon, rather than the amino nitrogen, appears to act as the nucleophile to attack an oxocarbenium phosphoribosyl intermediate formed after the loss of PP_i_ from PRPP. The proposed mechanism also predicts that PRPP binding may induce a protein conformational change that is critical for catalysis.

Kinetics studies using *N*-substituted analogs of *p*ABA have revealed that selected substrate analogs can inhibit RFAP synthase activity *in vitro* with *K_i_* values as low as 7.5 µM [13]. This work has provided the basis for designing agrichemicals that may decrease the levels of methane as a greenhouse gas emitted from microorganisms in the digestive tracts of domestic livestock such as cattle [13]. Interestingly, an abundance of methane-producing microorganisms in the digestive tracts if humans and other animals has also been correlated with obesity [14],[15]. Based on these studies, increased knowledge of the *p*ABA-binding site of RFAP synthase and inhibition of RFAP synthase activity by *N*-substituted *p*ABA analogs may deepen our understanding of microbial factors that contribute to obesity in humans.

At present, only limited information is available regarding the structure of RFAP synthase and the roles that specific amino acids play in substrate binding and catalysis. Computational molecular modeling of RFAP synthase from *M. jannaschii* has predicted that the substrate binding site lies at the interface between identical subunits of the RFAP synthase homodimer [12]. In a separate study, alanine-screening mutagenesis of RFAP synthase from the moderate thermophile *Methanothermobacter thermautotrophicus* (MTH0830) has identified several charged amino acids that may play roles in substrate binding or catalysis [16]. In particular, the substitution of alanine for aspartic acid at position 19 (D19A) or arginine at position 26 (R26A) resulted in the production of inactive protein. However, due to the lack of detectable enzyme activity in the altered proteins, the specific functions of residues D19 and R26 were not further investigated.

The purified MTH0830 enzyme has shown optimal activity at pH 7, nearly two pH units higher than the optimal pH values for RFAP synthases from *Archaeoglobus fulgidus* and *M. jannaschii* [3],[12],[17]. This difference in pH optimum is likely to alter the *K_M_* for the charged substrate pABA. In this work, to provide insight into specific amino acids required for RFAP synthase activity, site-directed mutagenesis was used to produce altered enzymes of the *M. thermautotrophicus* RFAP synthase with more conservative substitutions (D19N and R26K), and the wild-type and altered proteins were analyzed by enzymatic assay, isothermal titration calorimetry, and computational modeling.

## 2. Materials and methods

### 2.1. Chemicals

Restriction enzymes and T4 DNA ligase were purchased from New England Biolabs (Beverly, MA). *Pfu* DNA polymerase was from Stratagene (Agilent Technologies, Santa Clara, CA). Luria-Bertani broth mix (Becton Dickinson, Franklin Lakes, NJ) was purchased from Fisher Scientific (Suwanee, GA). Deoxyribonuclease I, citrate, MgCl_2_, 4-aminobenzoic acid (*p*ABA), 5-phospho-D-ribose 1-diphosphate sodium salt (PRPP) and *N*-(1-naphthyl) ethylenediamine were purchased from Sigma-Aldrich Chemical Company, Inc. (St. Louis, MO). Bradford protein assay reagent and polyacrylamide gels were from Bio-Rad (Hercules, CA). Gases were obtained from Strate Welding (Gainesville, FL) and Oxygen Services (Orange, CA). All other chemicals were of reagent grade and purchased from Fisher Scientific.

### 2.2. Recombinant expression of the MTH0830 RFAP synthase genes in E. coli

*MTH0830* has been previously identified as the gene encoding the H_4_MPT biosynthetic enzyme RFAP synthase in the methanogen *M. thermautotrophicus* [3]. For the heterologous production of the enzyme, cells of the *E. coli* expression strain KB1 [BL21(DE3) cells with the chaperone-encoding plasmid pG-Tf2 and the *MTH0830* expression plasmid pED2] [17] were grown, induced with 1 mM isopropylthiogalactoside, and harvested as described previously [17]. For expression of RFAP synthase variants, plasmids with the mutated genes were transformed as described for the wild-type into BL21(DE3) cells containing plasmid pG-Tf2 [17].

### 2.3. Site-directed mutagenesis of recombinant RFAP synthase

Plasmid pED2 containing the RFAP synthase gene (*MTH0830*) (17) was purified using the Spin MiniPrep plasmid isolation kit from Qiagen (Valencia, CA) and was used as the template for site-directed mutagenesis. Residues R26 and D19 of the *M. thermautotrophicus* RFAP synthase were altered in a non-conservative and conservative manner using the QuikChange XL kit (Stratagene, Agilent Technologies, Santa Clara, CA). The thermocycling conditions were denaturation at 95 °C for 30 s, primer annealing at 55 °C for 60 s, and extension for 12 min at 68 °C through 16 cycles. The sequences of the forward primers were 5′-CTCAACGGTGAGAGGGGCAAGCTTGACGGTGGAGTTGG for R26K and 5′-CCACCTGACCCTCATAAACCTCAACGGTGAGAGGG for D19N. Dideoxy sequencing [18] was used to verify the mutations (Interdisciplinary Center for Biotechnology Research, University of Florida, Gainesville, FL). The plasmids with the mutated genes were transferred into XL-10 Gold cells, purified, and used to transform competent BL21(DE3) cells containing the chaperone plasmid pG-Tf2. The cells were grown, and the *MTH0830* variant genes were expressed in the same manner as the wild type.

### 2.4. Purification of recombinant wild-type RFAP synthase

All purification steps were performed at ambient temperature in the presence of 2 mM dithiothreitol (DTT) or 15 mM beta-mercaptoethanol. Cells were lysed using a French press at 20,000 psi, and the cell lysate was centrifuged at 31,000 x g for 1 hour to separate the cell-free extract (CFE, supernatant) from the insoluble fraction (pellet).

For wild-type protein, CFE (50 mL) was heat-treated at 65°C for 15 min and centrifuged at 13,000 x *g* for 15 min to remove precipitated proteins. Heat-treated CFE (25 mL) was loaded onto a 40-mL ceramic hydroxyapatite column (2.5 × 11 cm) equilibrated with 50 mM TES [*N*-tris (hydroxymethyl) methyl-2-aminoethanesulfonic acid], 2 mM DTT, pH 6.8. The column was washed with 80 mL of the same buffer, and proteins were eluted using a 400 mL linear gradient from 0 to 125 mM NaH_2_PO_4_ in equilibration buffer at pH 6.8 collecting 20 mL fractions. Active RFAP synthase was found in fractions eluting between 60 and 80 mM NaH_2_PO_4_.

The most active hydroxyapatite fractions were loaded onto a Mono Q HR 5/5 anion exchange column (Amersham-Pharmacia Biotech-Piscataway, NJ) equilibrated with 50 mM TES, 5 mM MgCl_2_, 2 mM DTT pH 6.8. The column was washed with 300 mM NaCl in the equilibration buffer, followed by a 20 mL linear gradient from 300 mM to 600 mM NaCl. Active RFAP synthase was found in fractions eluting between 375 and 390 mM NaCl. The active Mono Q fractions were combined, concentrated, and loaded onto a Superdex 75 gel filtration column equilibrated with 50 mM TES, 150 mM KCl, and 2 mM DTT. The active pure RFAP synthase enzyme eluted after 19 min corresponding to a molecular mass of 68.7 kDa.

### 2.5. Determination of optimal enzyme assay conditions

RFAP synthase activity was determined by producing the azo-dye derivative of the product RFAP as described previously [1],[17]. To determine the temperature optimum, the reaction velocity of hydroxyapatite-purified enzyme was measured using 35 mM *p*ABA, 8.8 mM PRPP and 30 mM MgCl_2_, 150 mM NaCl, 50 mM MES (pH 6.8), at temperatures ranging from 37 °C to 80 °C. To determine the effect of enzyme concentration on the activity of RFAP synthase, the activity of enzyme (approximately 95% pure) was determined using 35 mM *p*ABA and 8.8 mM PRPP with various amounts of enzyme ranging from 0.012 to 0.200 nmol (1 to 25 µg). From this point on, the amount of enzyme used in activity assays was within the linear range of 8 to 12 µg. For determination of the *K_M_* for *p*ABA, the PRPP concentration was kept constant at 8.8 mM with 17.6 mM MgCl_2_, while the *p*ABA concentration was varied from 0.18 to 35 mM. All assays were carried out under optimal enzymatic conditions in 70 mM PIPES (pH 6.8), 2 mM DTT at 70 °C for 1 h.

### 2.6. Partial purification of wild-type RFAP synthase and variants for kinetic comparison

The RFAP synthase variants showed less heat stability than the wild-type enzyme. Therefore, for comparison, the variant and wild-type enzymes were partially purified from CFE (approximately 500 mg protein) by hydroxyapatite chromatography without heat treatment. CFE was loaded onto a 10 ml ceramic hydroxyapatite column equilibrated with 50 mM TES (pH 6.8), 2 mM DTT buffer and washed with 20 ml of the same buffer. A 100 ml linear gradient was applied to the column from 0 mM to 400 mM NaH_2_PO_4_ in equilibration buffer at pH 6.8, and 5 ml fractions were collected. Active wild-type RFAP synthase eluted between 150 mM and 200 mM NaH_2_PO_4_, while active variants eluted between 80 mM and 100 mM NaH_2_PO_4_.

The most active hydroxyapatite fractions were loaded onto a Mono Q HR 5/5 anion exchange column equilibrated with 50 mM TES, 5 mM MgCl_2_, 2 mM DTT pH 6.8, and the column was washed with 130 mM NaCl in the equilibration buffer, followed by a 30 ml linear gradient from 130 to 330 mM NaCl. Active RFAP synthase variants eluted between 120 and 130 mM NaCl. For comparison with the variants altered by site-directed mutagenesis, the recombinant wild type enzyme was partially purified using this modified protocol.

### 2.7. Kinetic analyses of recombinant RFAP synthase variants

To determine the *K_M_* for *p*ABA for the RFAP synthase variants using the enzyme assay, the specific activities of the Mono Q purified proteins were determined in the presence of 8.8 mM PRPP and 17.6 mM MgCl_2_, with a range of *p*ABA concentrations from 181 µM to 67 mM for the D19N variant, and 3.8 mM to 130 mM for the R26K variant. Due to the inability of the variants to function at 70 °C, the enzymatic assays comparing variant and wild-type kinetics were run in 70 mM PIPES (pH 6.8) with 2 mM DTT at 50 °C for three hours using a single time-point assay. This is the time period over which the enzyme activities were linear at the lower temperature of 50 °C. For these kinetic studies, the wild-type enzyme was purified in the same manner as the variants and was also examined at 50 °C for three hours.

### 2.8. Gel electrophoresis and protein concentration measurements

Protein purity was determined using reducing sodium dodecyl sulfate polyacrylamide gel electrophoresis (SDS-PAGE) [19] with Coomassie Brilliant Blue R-250 as the stain (Bio-Rad). Protein concentrations were determined using the Bradford protein assay (Bio-Rad, Hercules, CA) [20] with bovine serum albumin as the standard.

### 2.9. Isothermal titration calorimetry (ITC) of wild type and variant RFAP synthases

The ITC conditions previously established for *M. jannaschii* RFAP synthase [12] were modified for the RFAP synthase from *M. thermautotrophicus*. Partially purified wild-type (heat-treated at 65 °C) and variant (heat-treated at 50 °C) RFAP synthases (2–5 mL) were dialyzed against two changes of buffer (250 mL each of 50 mM Tris, 100 mM NaCl, pH 7.6). The dialysis buffer was used to create a 200 mM PRPP stock solution or a 48 to 200 mM *p*ABA solution. All ITC measurements were carried out using a *NANO-*ITC isothermal titration calorimeter (TA Instruments, New Castle, DE).

For determination of the *K_D_* for PRPP, a 20 µM solution of dialyzed RFAP synthase (250 µL) was equilibrated in the sample cell at 35 °C, and 50 µL of 200 mM PRPP was loaded into the injection syringe. Following an 1800 s delay, 10 injections of 5 µL of PRPP each were added every 240 seconds with stirring at 300 rpm. Because a previous study demonstrated ordered binding with PRPP binding first [12], *p*ABA binding in this work was measured in the presence of a saturating concentration of PRPP. For *p*ABA binding, the sample cell was equilibrated at 35 °C with 200 µL of 20 µM dialyzed RFAP synthase and 50 µL of 200 mM PRPP. For the wild-type enzyme, 48 mM *p*ABA was added in ten 5 µL aliquots to a total of 50 µL. For the R26K variant, the stock solution of *p*ABA was 200 mM. The binding constants for *p*ABA and PRPP were estimated by plotting the area under each peak of the resultant heat profile against the concentration of *p*ABA or PRPP and by fitting to a theoretical curve using the NanoAnalyze software provided with the instrument (TA instruments, New Castle, NE).

### 2.10. Molecular modeling of RFAP synthase

All molecular modeling and docking were performed using a stochastic global energy optimization procedure in Internal Coordinate Mechanics (ICM) using the MolSoft ICM-Pro package version 3.7 (Molsoft, San Diego, CA) [21],[22]. An alignment was generated between the *M. thermautotrophicus* RFAP synthase and the sequence of homoserine kinase using an adaptation of the Needleman and Wunsch algorithm [23],[24]. Homoserine kinase, the closest available homologous crystal structure in the Protein Databank (PDB 1FWK), was previously predicted to be a good model template for RFAP synthase from *M. jannaschii* [12].

The initial RFAP synthase model was produced by threading the sequence of *M. thermautotrophicus* RFAP synthase onto the template crystal structure of the homoserine kinase (PDB code: 1FWK subunit A). The model was refined by globally optimizing the side-chains and annealing the backbone. The iterative refinement procedure contained three main steps: (i) random sampling of the dihedral angles according to the biased probability Monte Carlo method [22], (ii) a local minimization step, and (iii) application of the Metropolis criterion [25] to accept or reject a conformation. A non-homologous loop region in the vicinity of the predicted ligand binding region (residues 23 to 30 of RFAP synthase) was modeled by searching a database of loop fragments to identify loop templates with matching ends and homologous sequence and refining the subsequent model. The final model was selected based on an ICM calculated energy profile [22],[26].

The MTH0830 RFAP ligand binding pocket was calculated using the ICMPocketFinder algorithm [27], and the pocket was analyzed using ICM-Pro. The PRPP ligand was docked to the pocket using the ICM-Docking method [21],[22].

## 3. Results

### 3.1. Purification of M. thermautotrophicus RFAP synthase produced in E. coli

**Figure 2.**
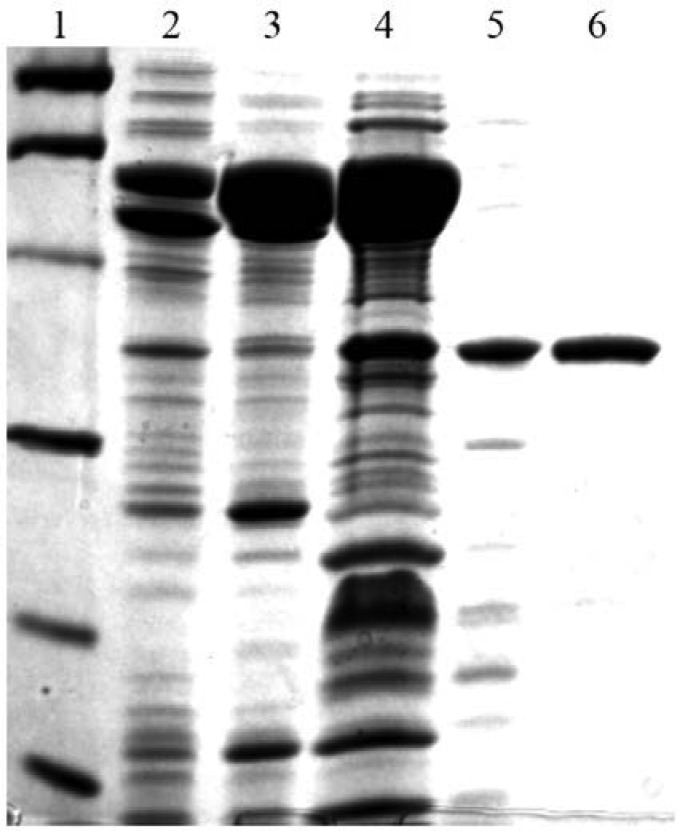
SDS-PAGE gel of RFAP synthase purification. Protein samples were boiled in the presence of 7.5% 2-mercaptoethanol in SDS-PAGE sample buffer and loaded onto a 12% polyacrylamide gel. The gel was stained with Coomassie Brilliant Blue R-250 (Bio-Rad). Lane 1, Molecular mass markers. Lane 2, CFE (8 µg). Lane 3, heated CFE (10.2 µg). Lane 4, hydroxyapatite fraction (17 µg). Lane 5, Mono Q fraction (2 µg). Lane 6, Superdex 75 fraction (2 µg). The bands in lane 1 indicate the positions of the following molecular mass markers: phosphorylase b (97.4 kDa), bovine serum albumin (66.2 kDa), ovalbumin (45 kDa), carbonic anhydrase (31 kDa), soybean trypsin inhibitor (21.5 kDa), and lysozyme (14.4 kDa).

In this work, a recombinant form of *M. thermautotrophicus* RFAP synthase was purified for analysis by enzyme assay, site-directed mutagenesis, and isothermal titration calorimetry. The enzyme was purified 2800 fold to homogeneity with a specific activity of 360 nmol/min/mg of enzyme (Table 1 and Figure 2). The 20 fold increase in specific activity following the Mono Q step (Table 1) may be due to the removal of the abundant chaperone proteins (Figure 2), which could have detrimentally affected the specific activity by binding to RFAP synthase in the early purification steps. Based on gel filtration using Superdex 75 medium and SDS-PAGE analysis, the native molecular mass of the purified enzyme was estimated to be 69 kDa, consistent with a homodimer of 38 kDa subunits.

**Table 1.** Purification of wild-type *M. thermautotrophicus* RFAP synthase produced in *E. coli*.

Purification step	Total activity^1^ (nmol per min)	Specific activity Yield (nmol per min per mg)	Fold (%)	purification
Cell-free extract	690	0.13	10	1
Heat-treated	390	0.79	57	6
Hydroxyapatite	260	14	38	110
Mono Q	110	290	16	2200
Superdex 75	30	360	4.2	2800

RFAP synthase assays were performed at 70 °C at pH 6.8 for one hour as described in the Material and Methods

### 3.2. Determination of optimal enzymatic conditions

To determine the optimal enzymatic conditions for recombinant methanogen RFAP synthase, the activity of hydroxyapatite-purified RFAP synthase was tested at different temperatures and pH values. The highest enzyme activity was found at 70 °C and pH 7.0, and the optimal MgCl_2_ concentration was 1.5 to 3 fold higher than the PRPP concentration used (data not shown). The activity of hydroxyapatite-purified RFAP synthase remained constant up to an ionic strength of 200 mM, and thus from this point, the activity of wild-type *M. thermautotrophicus* RFAP synthase was measured using these optimal conditions. For enzyme concentration, a non-linear concave relationship was observed between the amount of enzyme used and the reaction velocity until about 0.1 nmol (6.5 µg) of dimeric enzyme, at which point the relationship became linear until at least 0.2 nmol of enzyme (data not shown).

### 3.3. Kinetic analyses of recombinant RFAP synthases

**Figure 3.**
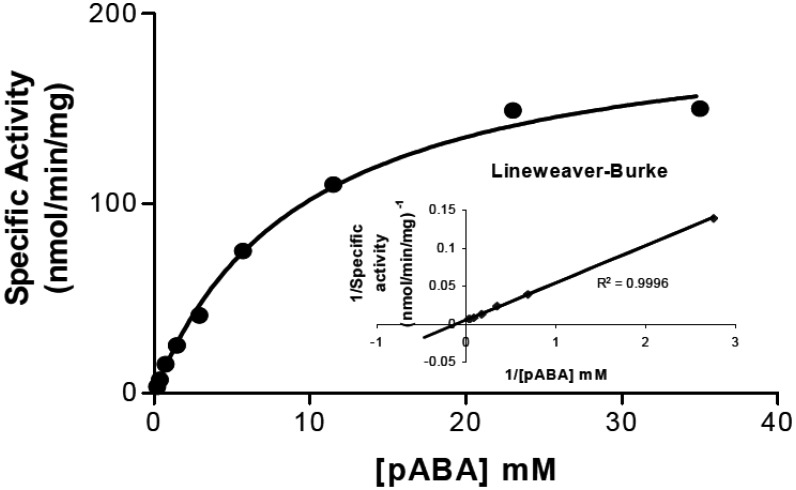
Determination of pABA *K_M_* for recombinant RFAP synthase. RFAP synthase activity was measured at a constant PRPP concentration of 8.8 mM while varying the pABA concentration from 181 µM to 36 mM. The inset shows the Lineweaver-Burke plot derived from the Michaelis-Menton plot. The estimated *K_M_* for pABA was 4.5 mM and the *V_max_* at 70 °C was approximately 190 nmol/min/mg protein.

During the course of the purification and biochemical analysis of the recombinant enzyme, it was observed that purification of the enzyme beyond the Mono Q anion exchange step (about 80% pure) resulted in loss of enzyme stability during storage. Thus, the *K_M_* for the substrate *p*ABA was measured using enzyme purified through the Mono Q step. When the enzyme activity was measured at 8.8 mM PRPP and 17.6 mM MgCl_2_, the *K_M_* for *p*ABA was estimated to be approximately 4.5 mM (Figure 3).

### 3.4. Bioinformatic analysis of RFAP synthase

**Figure 4.**
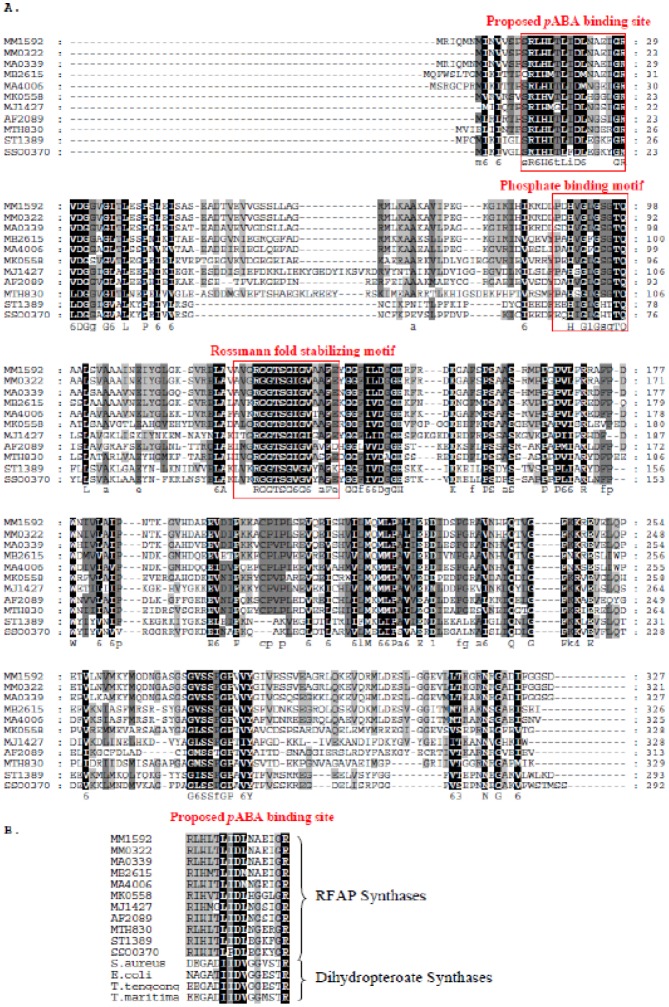
Bioinformatic analysis of group I RFAP synthase genes. A. Multiple amino acid sequence alignment of Group I RFAP synthases using the alignment program GeneDoc [43]. Possible phosphate binding motifs are indicated by brackets. The protein abbreviations are as follows: *Methanosarcina mazei*, MM1592 and MM0322; *Methanosarcina acetivorans*, MA4006 and MA0339; *Methanosarcina barkeri*, MB2615; *Methanopyrus kandleri*, MK0558; *Methanocaldococcus jannaschii*, MJ1427; *Archaeoglobus fulgidus*, AF2089; *Methanothermobacter thermautotrophicus*, MTH0830; *Sulfolobus tokodaii*, ST1329; *Sulfolobus sulfataricus*, SSO0370. B. Multiple amino acid sequence alignment showing homology between the conserved region of RFAP synthase and a predicted pABA-binding region of dihydropteroate synthase (DHPS) from *Staphylococcus aureus*, *Escherichia coli*; *Thermoanaerobacter tengcongensis*, and *Thermotoga maritima*.

RFAP synthase homologs have previously been divided into three groups based on phylogenetic analysis [3]. Group 1 homologs, including RFAP synthases from methanogenic archaea and *Sulfolobus*, share conserved regions that are less prominent in the remaining two groups. Groups 2 and 3 include other non-methanogenic archaea and the bacterial sequence from *Methylobacterium extorquens*. Multiple sequence alignment of group 1 RFAP synthases (Figure 4A) has shown two conserved regions with significant similarity to glycine-rich amino acid motifs that bind phosphoribosyl-containing substrates [28],[29]. The first region has high homology to the phosphate binding loop of mevalonate kinase, which is usually composed of a loop with the consensus sequence PX_3_GLGSSAA connecting a β-strand to an α-helix [30]. In mevalonate kinase, this binding loop is also known to interact with the pyrophosphate tail of ATP [28],[30]. The presence of a similar region in the RFAP synthase sequences suggests a role for this conserved region in binding the pyrophosphate of PRPP.

The second highly conserved region has significant homology to the consensus sequence [V/I]XGX_1-2_GX_2_GX_3_[G/A], known to stabilize β-strand and α-helix interactions for Rossmann fold proteins that bind FAD and NAD(P) (Figure 4A) [29]. In these proteins the β-strand/α-helix loop not only stabilizes the Rossmann fold but also interacts with the nucleotide near the phosphoribosyl moiety. The presence of this sequence in RFAP synthase homologs provides evidence that this region likely binds to the phosphoribosyl portion of PRPP and possibly works together with the first putative phosphate binding loop to form an overall PRPP binding region within the active site of the enzyme. It is interesting to note however, that this particular conserved region is not present in the groups 2 and 3 RFAP synthase homologs [31].

A third conserved region (amino acids 11 to 26 in *M. thermautotrophicus*) has a number of charged residues, including D19 and R26 (Figure 4A and 4B). Bioinformatic analysis showed that these amino acids share up to 40% identity with a region of the enzyme dihydropteroate synthase that forms part of a channel that binds *p*ABA [32]. These similarities provided support for the proposal that the region containing D19 and R26 in RFAP synthase may play a role in binding *p*ABA.

### 3.5. Site-directed mutagenesis

In a previous study, to begin to identify amino acid residues critical for substrate binding and catalysis, RFAP synthase from *M. thermautotrophicus* (MTH0830) was studied using alanine screening mutagenesis of highly conserved charged amino acids, including arginine 26 (R26) and aspartic acid 19 (D19) [16]. Two variants (R26A and D19A) produced in *E. coli* were partially purified, revealing that R26A and D19A were soluble, but lacked detectable RFAP synthase activity. The non-conservative replacement of R26 and D19 with alanine removed the charged functional groups, which may explain the lack of activity in spite of the production of soluble protein. In the current work, more conservative substitutions of R26 and D19 were constructed. R26 was replaced with a lysine (R26K) to maintain a positive charge on the side chain, and D19 was replaced with an asparagine (D19N) to maintain side chain polarity.

Initial characterization of the R26K and D19N variants showed that they were soluble and had low, but detectable, enzyme activity at 70 °C, which is the temperature optimum for the wild-type enzyme. The temperature optima for the R26K and D19N variants, however, were found to be significantly lower (50 °C and 60 °C, respectively) than that of the wild-type. Thus, for consistency, the activities of the variant and wild-type enzymes were studied at 50 °C. At this temperature, the wild-type enzyme purified by hydroxyapatite and Mono Q chromatography (without the 65 °C heat-step), exhibited an apparent *K_M_* for *p*ABA of approximately 3.9 mM at 50 °C, similar to the wild-type value obtained at 70 °C (4.5 mM). However, the apparent *V_max_* for the wild-type enzyme at 50 °C (1.45 nmol/min/mg protein) was lower, most likely due to the lower temperature used and partial purification (Table 2).

**Table 2.** Kinetic values determined for wild-type and altered RFAP synthases assayed at 50 °C.

Conservative substitution	Temperatureoptimum (°C)	*K_M_* for pABA^1^(mM)	*V_max_*^1^(nmol/min/mg)	
Wild-type	70	3.9	1.5	
R26K	50	38	5.0	
D19N	60	12	0.5	

Assays for *K_M_* determination were conducted at 50 °C for 3 h.

When the R26K and D19N variants were partially purified in the same way and assayed at 50 °C, R26K showed an apparent *K_M_* for pABA of 38 mM, which is nearly ten-fold higher than the *K_M_* for the wild-type under similar conditions (Table 2). This result would be consistent with a role for R26 in the binding of *p*ABA. Under the conditions studied, the *V_max_* for the R26K variant was approximately 5.0 nmol/min/mg, about 3-fold higher than that of the wild-type.

The estimated *K_M_* for the D19N variant was 12 mM, which is three-fold higher than the wild-type, while the *V_max_* for the D19N variant was approximately 0.45 nmol/min/mg (Table 2). The higher *K_M_* might also be consistent with a role for D19 in the binding of *p*ABA, although D19 would appear to play a more subtle role than R26 in substrate binding. The differences between the wild-type and variant *V_max_* values could suggest that altering the amino acids may slightly affect the catalytic properties as well.

### 3.6. Isothermal titration calorimetry (ITC)

Given the modest three-fold effect of the D19N alteration on the *K_M_* for *p*ABA, we sought an additional means of assessing the effect of the asparagine substitution on *p*ABA binding. It was also of interest to determine the effect of the amino acid substitutions on the binding constant for PRPP. Previously, Dumitru and Ragsdale [12] used ITC to determine the *K_D_* values for PRPP binding to the hyperthermophilic RFAP synthase from the *M. jannaschii*. An advantage of this technique is that ITC can measure the heat released upon binding of individual substrates, even if the enzyme is catalytically incompetent.

Using ITC, the estimated *K_D_* values of PRPP and pABA were 2.1 mM and 0.96 mM, respectively, for the wild-type MTH0830 RFAP synthase. Based on triplicate ITC determinations, the estimated *K_D_* for PRPP binding to the D19N variant was 2.0 mM, similar to the wild type value of 2.1 mM (Table 3) and to the value obtained previously for RFAP synthase from *M. jannaschii* [12]. This result indicates that replacing the carboxylic acid side chain of D19 with an amide has no detectable effect on PRPP binding. In contrast, the apparent *K*_D_ for *p*ABA was (3.7 mM), about 4 times higher than the *K*_D_ of the wild type enzyme (0.96 mM). This four-fold increase in the *K_D_* for *p*ABA estimated by ITC is similar to the three-fold increase in *K_M_* estimated by enzymatic assay (Table 2) and provides evidence for only a subtle role of D19 in *p*ABA binding.

**Table 3.** Isothermal titration calorimetry of wild type and site-directed variants of MTH0830 RFAP synthase.

RFAP Synthase Variant	*K_D_* for PRPP (mM)	Apparent *K_D_* for *p*ABA (mM)
Wild-type	2.1 ± 0.11	0.96 ± 0.65
D19N	2.0 ± 0.30	3.7 ± 0.45
R26K	2.4 ± 0.25	24 ± 2.2

For R26K, the estimated *K_D_* for PRPP using ITC was 2.4 mM, which was comparable to the wild type *K_D_* of 2.1 mM (Table 3). However, the apparent *K*_D_ for pABA (24 mM) was approximately 25 times higher than that of the wild-type under the ITC conditions. These data are consistent with the results using enzymatic assays (Table 2), which showed a dramatic increase in *K_M_* when R26 was substituted with lysine. Combined with the previous report that the R26A variant shows no detectable enzyme activity, these results indicate that the positive charge of the R26 side chain may play an important function in the binding of *p*ABA.

### 3.7. Molecular modeling of M. thermautotrophicus RFAP synthase (MTH0830)

**Figure 5.**
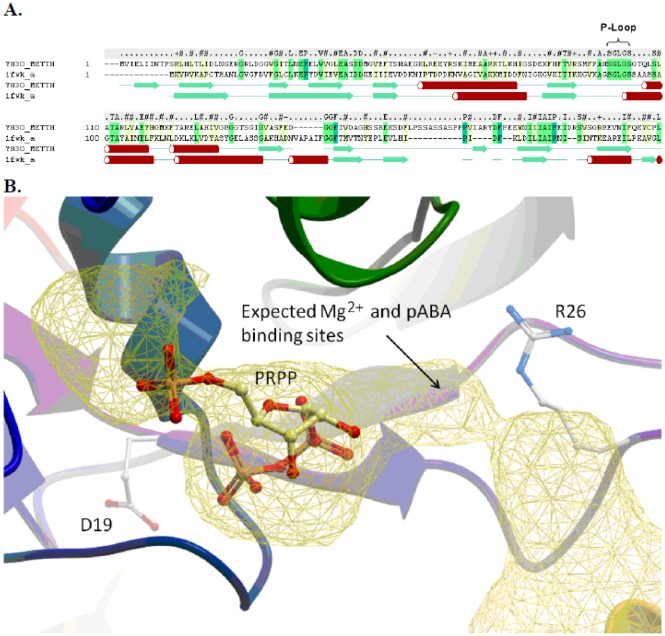
Computational molecular model of MTH0830 RFAP synthase. A: Secondary structure and sequence alignment of the N-terminus of MTH0830 (Y830_METTH) (from residue 1 to 215) and homoserine kinase template structure (pdb code: 1fwk) [44]. The secondary structure elements are colored green (beta Sheet) and red (alpha helix). The secondary structure of MTH0830 was calculated using the DSSP method [45], and the secondary structure of 1fwk is shown as defined in the Protein Data Bank file. B: The predicted PRPP and pABA binding pocket. The MTH0830 model is displayed in ribbon colored from the N to C terminus (blue – red), residues D19 and R26 are displayed as a stick representation. The docked PRPP ligand is displayed in stick representation with carbon atoms colored yellow. The binding pocket is displayed as a yellow mesh, and the predicted sites for *p*ABA and Mg^2+^ are annotated.

The crystal structure for homoserine kinase was used previously as the template to generate an overall model for RFAP synthase from *M. jannaschii* [12]. To gain additional insight into the potential roles of R26 and D19 in *p*ABA binding in MTH0830, a molecular model of RFAP synthase from *M. thermautotrophicus* was generated by threading the MTH0830 sequence onto the crystal structure of homoserine kinase from *M. jannaschii* complexed with ADP (PDB 1FWK) (Figure 5).

Comparison of the amino acid sequences of MTH0830 and homoserine kinase showed 36% similarity and 21% identity. The suitability of this template for modeling was further supported by (i) the predicted secondary structure of MTH0830 RFAP synthase which showed good alignment with homoserine kinase in the N-terminal region and (ii) perfect conservation in the SGLGS sequence corresponding to the P-Loop motif of nucleotide binding proteins (Figure 5A).

The previous model of RFAP synthase from *M. jannaschii* [12] predicted that the active site of the homodimer is located at the interface between the two subunits. Figure 5B illustrates the predicted active site for one subunit of the *M. thermautotrophicus* RFAP synthase.

Secondary structure analysis predicted that D19 occurs at the C-terminal end of a beta sheet, while the region surrounding R26 (residues 23 to 30) forms a non-homologous loop, which was modeled by searching a database of loop fragments and identifying loop templates with matching ends and homologous sequence followed by refinement.

Figure 5B shows the most energetically favorable pose for PRPP in the substrate binding pocket. PRPP was predicted to interact noncovalently with the side chains of lysine 69 and histidine 98 as well as the backbone of histidine 98 and residues 101 to 104. The latter two amino acids are part of a conserved phosphate-binding loop motif previously identified among RFAP synthases (Figure 4).

In the molecular model (Figure 5B), R26 occupies a key position in the predicted *p*ABA-binding pocket on the side distal from PRPP. R26 has no interaction with PRPP, but is in a position to interact with *p*ABA possibly by forming a salt bridge. The formation of a salt bridge with arginine or lysine could explain why the alanine variant (R26A) is inactive and unable to bind *p*ABA [16]. In contrast, R26K retains partial *p*ABA-binding ability and catalytic competence.

With PRPP docked to the RFAP synthase monomer, D19 appears to occupy a position in the active site where it is unlikely to make direct contact with *p*ABA. As no specific interaction with *p*ABA could be predicted, it is possible that D19 may participate in the overall structure or conformational shape of the *p*ABA binding pocket without affecting PRPP binding affinity. This positioning could also be indicative of the protein conformational change predicted previously for the Bi-Ter kinetic mechanism and/or the formation of the active site by the interaction of two subunits [12].

## 4. Discussion

The formation of RFAP from *p*ABA and PRPP is the committed step in the H_4_MPT biosynthetic pathway. Moreover, the resulting decarboxylation of *p*ABA by RFAP synthase produces the chemical feature that distinguishes H_4_MPT from tetrahydrofolate [1],[11],[33]. Due to the low abundance of RFAP synthase present in methanogenic archaea [3],[17], it was necessary to develop a recombinant expression system for the RFAP synthase gene from *M. thermautotrophicus* (*MTH0830*) to further analyze the biochemical properties of the enzyme. Previous reports have suggested that inhibitors of RFAP synthase may help to mitigate the production of methane by methanogenic microorganisms in the digestive tracts of both livestock and humans, showing potential for future application to the development of future agrichemical and pharmaceutical chemicals [13],[14]. The homolog from the moderate thermophile *M. thermautotrophicus* was selected for the current studies because the growth temperature optimum for this organism is closer than the optima for RFAP synthases from hyperthermophiles to the growth temperature of animals [3],[12]. In addition, the complete genome for this methanogen is readily available [34], and alanine-screening mutagenesis has been previously carried out on the *M. thermautotrophicus* enzyme.

*M. thermautotrophicus* RFAP synthase was produced in *E. coli* and purified to homogeneity (Figure 2) with a final specific activity of 360 nmol/min/mg protein (Table 1). This value is slightly higher than the rates obtained for partially purified recombinant RFAP synthases from the hyperthermophilic archaea *M. jannaschii* (248 nmol/min/mg protein) [12] and *Archaeoglobus fulgidus* (290 nmol/min/mg protein) [3]. The purified recombinant *M. thermautotrophicus* RFAP synthase was a dimer of identical subunits when separated on a Superdex 75 gel filtration column, consistent with the quaternary structure reported for other active RFAP synthases [3],[12].

When the recombinant *M. thermautotrophicus* RFAP synthase was analyzed biochemically, some interesting differences emerged. In contrast to the hyperthermophilic enzymes, the enzyme from *M. thermautotrophicus* showed high activity at the physiological pH of 7, while the hyperthermophilic enzymes showed pH optima around pH 5 [3],[12]. For MTH0830, enzymatic assays estimated that the *K_M_* for *p*ABA was about 4 mM (Figure 3 and Table 2), and this value is substantially higher than the apparent *K_M_* for *p*ABA determined for RFAP synthases from *M. thermophila* and *M. jannaschii* (58 and 150 µM, respectively) [2],[12]. To rule out the possibility that the high *K_M_* value was an artifact of heterologous protein production in *E. coli*, native RFAP synthase was partially purified from the original organism *M. thermautotrophicus* (a gift from Dr. Ralph Wolfe, University of Illinois). The apparent *K_M_* for the native enzyme from *M. thermautotrophicus* cells was 3.9 mM (data not shown), similar to the recombinant enzyme, providing additional support that the *K_M_* for *p*ABA of the *M. thermautotrophicus* homolog is substantially different from previously studied methanogen RFAP synthases. The differences in pH optima may account in part for the higher *K_M_* for *p*ABA observed in the *M. thermautotrophicus* enzyme. The *K_M_* differences could reflect differing ionization environments within the active sites due to amino acid variation between the enzymes. Alternatively, since *p*ABA has two acidic pKa values of 4.65 and 4.80 [35], the higher *K_M_* for the *M. thermautotrophicus* enzyme at its pH optimum of 7, may indicate that the protonation state of either the amino or carboxylic acid group of *p*ABA plays an important role in substrate binding affinity. Our hypothesis is that the acidic (protonated) form of pABA, rather than the deprotonated form, is the actual substrate for the reaction. Thus, when we used a pH of 6.8 instead of 5.3 as for the previously published enzymes (2, 3, 12), a large proportion of the pABA would have been deprotonated. At near neutral pH, the concentration of protonated pABA would be lower by 1–2 orders of magnitude, resulting in a higher apparent *K_M_*. This is consistent with the 26 fold higher *K_M_* we observed for the *M. thermautotrophicus* enzyme under near neutral conditions when compared to previous homologs with a pH optimum at 5.3 (2, 3, 12). The effect of pH would be a significant consideration for inhibiting methanogenesis under physiological conditions in cattle rumen, where the pH is typically neutral.

Bioinformatic analysis of conserved regions among the RFAP synthase sequences revealed two phosphate-binding motifs, which are likely to interact with the phosphates of the substrate PRPP [30] (Figure 4A). A second region (amino acids 16 and 26) showed similarity to a predicted *p*ABA-binding site in dihydropteroate synthase (Figure 4B). The structure of dihydropteroate synthase is made up of a TIM-barrel fold composed of eight helices surrounding eight central β-strands with 8 loops connecting the α-helices and β-strands playing critical roles in catalysis [32]. In dihydropteroate synthase from *Mycobacterium tuberculosis*, it is thought that loop 2 aids in the overall formation of a *p*ABA binding site within a solvent inaccessible channel [32]. The similarity of the loop containing R26 in RFAP synthase to the loop 2 region of dihydropteroate synthase, including the presence of conserved arginine and aspartic acid residues (Figure 4), could indicate a similar role in *p*ABA binding for this region in RFAP synthase.

To test this hypothesis, site-directed mutagenesis of two conserved charged amino acids (R26 and D19) in the putative *p*ABA-binding region was undertaken. The non-conservative replacement of these residues to alanine was previously shown to produce soluble enzymes that are devoid of detectable enzyme activity [16], supporting a role for R26 and D19 in the activity of RFAP synthase. However, the inactivity of the variants did not allow for the determination of the specific role of these amino acids. In the current study, the more conservative substitutions of R26 with lysine (R26K) and D19 with asparagine (D19N) produced enzymes that retained partial activity. Based on isothermal calorimetry, the binding constant (*K_D_*) for PRPP for the wild-type, R26K, and D19N enzymes were similar to the value of 2 mM reported for the *M. jannaschii* enzyme [12]. In contrast, using either enzymatic assay or ITC, the *K_M_* for the second substrate *p*ABA increased at least 10 fold for R26K and 3–4 fold for D19N (Tables 2 and 3). These kinetic results are consistent with the prediction that R26 and D19 affect enzyme interactions with *p*ABA but not PRPP.

It is of interest to note that the apparent *K_D_* for wild-type *p*ABA binding obtained by ITC experiments (0.96 mM, Table 3) was lower than the *K_M_* determined using the enzymatic assay at pH 7 (3.9 mM, Table 2), but higher than the value obtained previously for RFAP synthase from *M. thermophila* (58–150 µM at pH 4.8) [2],[3]. We suspect two reasons for these differences. First, consistent with previous studies [12], the ITC studies were performed in the absence of the cofactor Mg^2+^, and the absence of this ion may have affected *p*ABA affinity. Second, the final pH of the ITC assays in this study was 6, intermediate between the pH values of 4.8 and 7.0 used in the enzymatic assays. Although the wild-type *K_M_* and *K*_D_ values for *p*ABA differ using the ITC versus enzymatic assays, both data show that substitution of R26 with lysine substantially lowered the affinity of the enzyme for *p*ABA without affecting the affinity for PRPP.

A model of MTH0830 RFAP synthase was generated by threading the sequence of MTH0830 RFAP synthase onto the template crystal structure of the homoserine kinase. Docking studies predicted that the PRPP binds first within the predicted active site where a key nucleotide binding motif is conserved (Figure 5). This is consistent with the Bi-Ter mechanism proposed for RFAP synthase from *M. jannaschii*, which predicts a requirement for PRPP binding prior to *p*ABA (12). Computational modeling of RFAP synthase predicted that the amino acid D19 is located close to the active site, but not directly interacting with it and somewhat distant from the predicted *p*ABA binding site. In this position, D19 could have an affect on the overall conformation of the binding pocket without interacting directly with *p*ABA. This can explain why a conservative change from aspartic acid to asparagine had only a subtle effect on the apparent *K_M_* or *K*_D_ of pABA. On the other hand, the model places the guanidinium of arginine 26 close to the predicted position for *p*ABA (Figure 5) in an orientation that could allow a noncovalent interaction with *p*ABA.

In dihydropteroate synthase [32],[36], the conserved arginine corresponding to R26 is predicted to contribute its guanidinium group for hydrogen bonding as well as its main chain for hydrophobic interactions during the binding of *p*ABA. In the computational model for RFAP synthase, R26 is in a position that allows hydrogen bonding with *p*ABA (Figure 5), which is supported by the site-directed mutagenesis results. While substituting R26 with alanine eliminated enzyme activity, conservative replacement with lysine (R26K) resulted in active RFAP synthase with substantially lower affinity for *p*ABA (Tables 2 and 3). The increase in the *K_M_* for *p*ABA for the R26K variant, along with the previously proposed role for the corresponding arginine in dihydropteroate synthase is consistent with a role for the amino group or positive charge of the guanidinium of R26 in *p*ABA binding.

In RFAP synthase, the replacement of D19 with asparagine resulted in the production of a soluble, active D19N variant with only a threefold increase in the *K_M_* for *p*ABA (Table 2). The small increase in the *K_M_* for *p*ABA suggests less of a direct role in substrate binding, but does not rule out a possible a role in catalysis or overall structure of the active site. The presence of a conserved carboxylate residue surrounded by hydrophobic residues has previously been proposed to be involved in the stabilization of a carboxonium ion intermediate formed during one of the proposed mechanisms of RFAP synthase, as well as in other phosphoribosyl transferase (PRTase) enzymes [2],[37].

Bioinformatic analysis of the RFAP sequences showed two phosphate-binding motifs. Distinct differences in the active site structures of PRTases have resulted in two evolutionarily distinct types of PRTases. The active site of type I PRTases are made up of an α/β core structure with a characteristic Rossmann fold and a solvent exposed active site that is flanked by hood and loop structures which close over the active site during catalysis [38]–[40]. The active structure of type II PRTases, solely represented by quinolinate phosphoribosyltransferase, is made up of a seven stranded α/β barrel open sandwich structure, which serves as a cap for the active site of the other subunit [38]. The data presented here along with the identification of a characteristic Rossmann fold and an apparent phosphate binding loop in RFAP synthase suggests a model for similar to that of type I PRTases [38]–[40]. The absence of this characteristic Rossmann fold in the two remaining groups of RFAP synthase homologs suggests not only structural differences, but also differences in substrate binding modes between the groups of RFAP synthases. This proposed model for the active site structure and catalytic mechanism of RFAP synthase, like that of other type I PRTases, would involve the formation of a slightly hydrophobic active site that would be inaccessible to bulk solvent water upon the sequential or random binding of the substrates [38]–[40]. The ability of the active site of RFAP synthase to form a solvent inaccessible active site would be crucial for the enzyme to catalyze the phosphoribosyl transfer event, as bulk solvent water can serve as a nucleophile hydrolyzing the activated substrate PRPP [32],[41],[42]. This model would also accommodate a previously proposed two-step S_N_1-like mechanism for RFAP synthase, which involves the formation of a carboxonium ion intermediate of PRPP that may be stabilized by carboxylate residues [12].

The work presented here contributes to the fundamental understanding of ligand-binding interactions in an important enzyme of biological methanogenesis. Increased knowledge of this enzyme could have multiple practical impacts for elucidating the structure and function of this enzyme and developing more specific RFAP synthase inhibitors to mitigate methane production related to agricultural production of greenhouse gases [13] as well as human health [14].
